# *Bifidobacterium mongoliense* genome seems particularly adapted to milk oligosaccharide digestion leading to production of antivirulent metabolites

**DOI:** 10.1186/s12866-020-01804-9

**Published:** 2020-05-07

**Authors:** Pauline Bondue, Christian Milani, Emilie Arnould, Marco Ventura, Georges Daube, Gisèle LaPointe, Véronique Delcenserie

**Affiliations:** 1 Department of Food Science, Fundamental and Applied Research for Animal and Health, Faculty of Veterinary Medicine, University of Liège, Liège, Belgium; 2 Laboratory of Probiogenomics, Department of Chemistry, Life Sciences and Environmental Sustainability, University of Parma, Parma, Italy; 3 Canadian Research Institute for Food Safety, University of Guelph, Guelph, Canada

**Keywords:** *Bifidobacterium mongoliense*, *Bifidobacterium crudilactis*, Whey, Bovine milk oligosaccharide, 3′-sialyllactose, Antivirulent effect, *Escherichia coli* O157:H7, *Salmonella enterica* serovar Typhimurium

## Abstract

**Background:**

Human milk oligosaccharides (HMO) could promote the growth of bifidobacteria, improving young children’s health. In addition, fermentation of carbohydrates by bifidobacteria can result in the production of metabolites presenting an antivirulent activity against intestinal pathogens. Bovine milk oligosaccharides (BMO), structurally similar to HMO, are found at high concentration in cow whey. This is particularly observed for 3′-sialyllactose (3′SL). This study focused on enzymes and transport systems involved in HMO/BMO metabolism contained in *B. crudilactis* and *B. mongoliense* genomes, two species from bovine milk origin. The ability of *B. mongoliense* to grow in media supplemented with whey or 3′SL was assessed. Next, the effects of cell-free spent media (CFSM) were tested against the virulence expression of *Escherichia coli* O157:H7 and *Salmonella enterica* serovar Typhimurium.

**Results:**

Due to the presence of genes encoding β-galactosidases, β-hexosaminidases, α-sialidases and α-fucosidases, *B. mongoliense* presents a genome more sophisticated and more adapted to the digestion of BMO/HMO than *B. crudilactis* (which contains only β-galactosidases). In addition, HMO/BMO digestion involves genes encoding oligosaccharide transport systems found in *B. mongoliense* but not in *B. crudilactis*. *B. mongoliense* seemed able to grow on media supplemented with whey or 3′SL as main source of carbon (8.3 ± 1.0 and 6.7 ± 0.3 log cfu/mL, respectively). CFSM obtained from whey resulted in a significant under-expression of *ler*, *fliC*, *luxS*, *stx1* and *qseA* genes (− 2.2, − 5.3, − 2.4, − 2.5 and − 4.8, respectively; *P* < 0.05) of *E. coli* O157:H7. CFSM from 3′SL resulted in a significant up-regulation of *luxS* (2.0; *P* < 0.05) gene and a down-regulation of *fliC* (− 5.0; *P* < 0.05) gene. CFSM obtained from whey resulted in significant up-regulations of *sopD* and *hil* genes (2.9 and 3.5, respectively; *P* < 0.05) of *S.* Typhimurium, while CFSM obtained from 3′SL fermentation down-regulated *hil* and *sopD* genes (− 2.7 and − 4.2, respectively; *P* < 0.05).

**Conclusion:**

From enzymes and transporters highlighted in the genome of *B. mongoliense* and its potential ability to metabolise 3′SL and whey, *B. mongoliense* seems well able to digest HMO/BMO. The exact nature of the metabolites contained in CFSM has to be identified still. These results suggest that BMO associated with *B. mongoliense* could be an interesting synbiotic formulation to maintain or restore intestinal health of young children.

## Background

The carbohydrate sources present in food influence microbiota composition and have an impact on young children’s health [1–3]. Simple and linear oligosaccharides, such as galacto-oligosaccharides (GOS) and fructo-oligosaccharides (FOS), are added to infant milk formula. They are bifidogenic but can be metabolised as well by other bacteria such as *Bacteroides* spp. or *Clostridium* spp., other bacteria found in the microbiota [4, 5]. In comparison, human milk is rich in complex sugars called human milk oligosaccharides (HMO). More than 500 structures have been identified and their concentration can reach up to 50 g/L or more in the colostrum [6] and 15 g/L in the mature milk [7]. The presence of α- and β-bonds protects them against digestion by the host and by most bacteria. A little quantity of HMO is absorbed across the epithelial barrier to reach the systemic circulation and then be excreted in the urine [8]. In addition, free milk oligosaccharides are able to decrease pathogen colonisation of intestinal cells by altering cell surface glycosylation and to increase bifidobacteria adherence [9].

In children’s faeces, the microbiota is dominated by *Bifidobacterium longum* (56.2%) and *Bifidobacterium bifidum* (10.7%) [10]. These two species, especially *Bifidobacterium longum* subsp. *infantis*, possess the enzymatic activity able to degrade these specific α- and β-bonds [11, 12]. In addition, the bifidobacteria proportion in breastfed children’s faeces can reach more than 90% [13]. In addition, bifidobacteria and lactic acid bacteria produce several metabolites inhibiting virulence gene expression of different pathogenic bacteria such as *Escherichia coli* O157:H7 [14, 15], *Salmonella enterica* serovar Typhimurium SA 941256 [16, 17] and *Campylobacter jejuni* [18].

Because they have a similar structure, bovine milk oligosaccharides (BMO) share common properties with HMO. More than 60 BMO have been identified [7] and a significant overlap between BMO and HMO has been demonstrated [19]. The BMO concentration in bovine milk is 20-fold lower than the HMO concentration in human milk [19]. In addition, the BMO degree of polymerisation is lower and fucosylated oligosaccharides are very poorly represented [19–22]. Like HMO, BMO present α- and β-bonds and are therefore protected against degradation by other bacteria [4]. With a concentration of 0.85 g/L in colostrum, the 3′-sialyllactose (3′SL: NeuAcα2–3Galβ1–4Glc) is one of the most important BMO found in cow milk [23, 24].

The whey, a by-product issued from the dairy industry, is obtained after the casein precipitation [25]. Its composition, depending on the method of cheese manufacture, is rich in protein such as β-lactoglobulin and α-lactalbumin, in lactose and in minerals [26]. The whey permeate (whey stripped of its large proteins after membrane filtration) contains the BMO found in the milk and may be considered as a potential source of prebiotics able to improve human health [25]. In addition, the high quantities of whey produced by the dairy industry could easily contribute to an oligosaccharides production at very large-scale, increasing its economic value [27]. The 3′SL is the most abundant oligosaccharide found in the whey permeate and its concentration in a whey issued from colostrum reached 94 g/L [28]. The consumption of BMO issued from whey is safe and well-tolerated [29] and its supplementation with an appropriate probiotic had a bifidogenic effect [30].

The epithelial adhesion of *B. longum* subsp. *infantis* ATCC 15697 was improved up to 9.8-fold to HT-29 cells in presence of 3′SL and 6′sialyllactose (6’SL) [31] and up to 3.3-fold in the presence of immunoglobulin G enriched from bovine whey oligosaccharides [32]. Zeinhom et al. [15] highlighted the protective effect of *Lactobacillus acidophilus* La-5 grown in medium supplemented with whey against enterohaemorrhagic *E. coli* (EHEC) infection in mice. Recently, Cooper et al. [33] demonstrated that a synbiotic combination of *B. animalis* subsp. *lactis* CNCM I-3446 and whey permeate containing 3′SL had a strong bifidogenic effect on microbiota of children who were born to an HIV+ mother.

Before reaching the colon to digest oligosaccharides, bifidobacteria must survive the stomach acidity, bile salts and pancreatic enzymatic activity. Most bifidobacteria do not support the presence of oxygen, making their production at industrial scale more challenging. Two bifidobacterial strains isolated from raw cow milk cheese, *Bifidobacterium mongoliense* FR/49/f/2 and *Bifidobacterium crudilactis* FR/62/b/3, are able to tolerate presence of oxygen and acidity [34, 35]. In addition, their genome could encode enzymes degrading BMO [36]. Therefore, these two strains could be an interesting source of probiotics for formula supplementation [35].

The first aim of this study was to determine the enzymes and transport systems involved in BMO degradation using the complete genome of *B. crudilactis* LMG 23609 and *B. mongoliense* DSM 21395. The second aim of this work was to evaluate the potential growth of *B. mongoliense* FR/49/f/2 in culture media supplemented with whey or 3′SL and to assess the effects of filtered supernatants on virulence expression of *E. coli* O157:H7 and *S.* Typhimurium.

## Results

### Enzymes and transporters involved in milk oligosaccharides

**Fig. 1:**
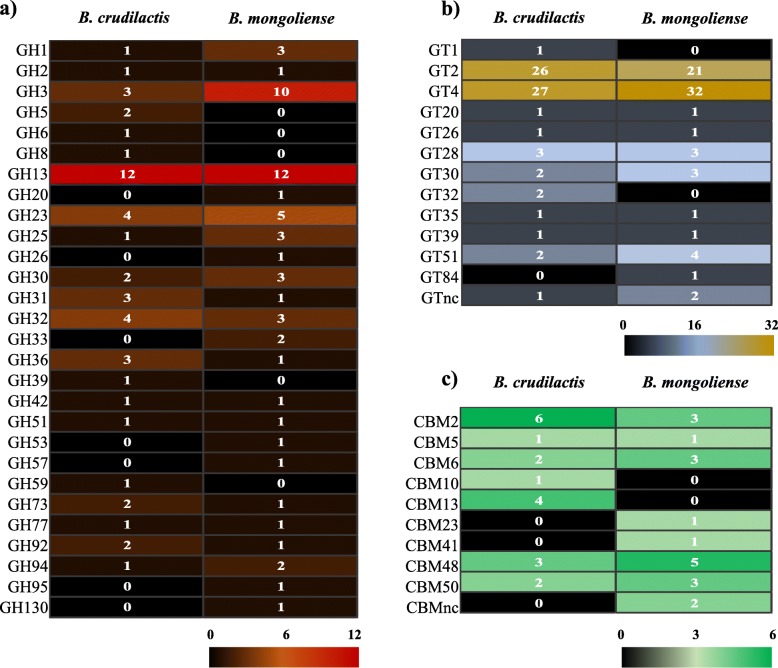
Enzymatic profiles involved in sugar degradation by *B. crudilactis* and *B. mongoliense*. Heat maps showing the number of genes identified for each glycoside hydrolases (GH) family (**a**), glycoside transferases (GT) family (**b**) and each carbohydrate-binding modules (CBM) family (**c**) in *B. crudilactis* and *B. mongoliense* genomes

**Fig. 2:**
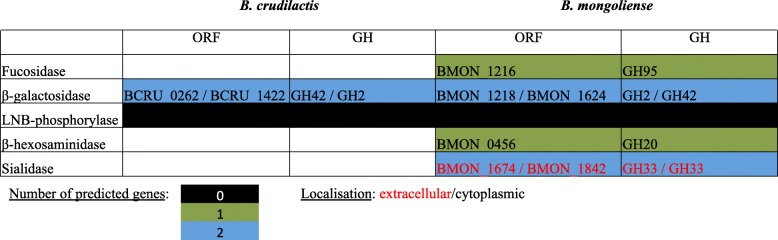
Digestion of milk oligosaccharides by *B. crudilactis* and *B. mongoliense*. The figure reports the presence/absence and CAZy classification of genes involved in milk oligosaccharides metabolism found in *B. crudilactis* and *B. mongoliense* genomes. Genes predicted to be intracellular are written in black while genes predicted to be extracellular are written in orange. ORF: open reading frame; GH: glycoside hydrolase

**Fig. 3:**
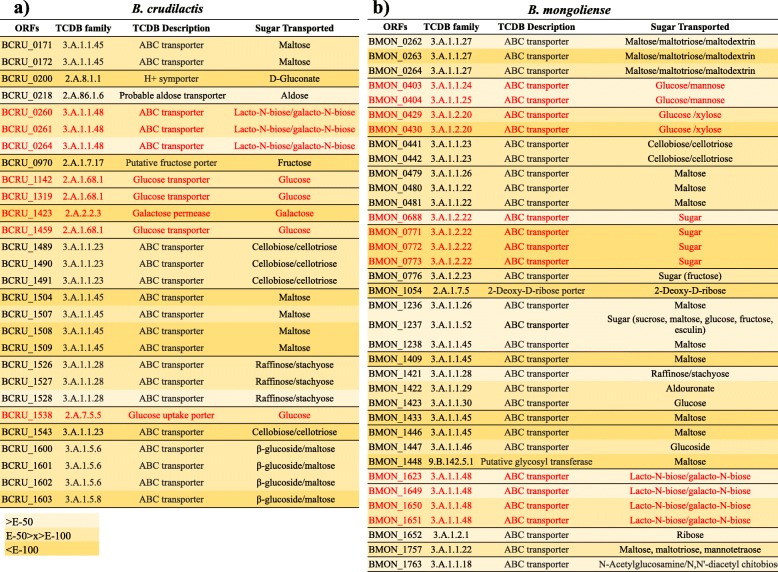
Sugar transporters identified in *B. crudilactis* (**a**) and *B. mongoliense* (**b**) genomes. Genes involved in milk sugars transport are indicated in red while the others are indicated in black. An E-value close to zero is indicated in dark yellow while an E-value far from zero is indicated in pale yellow. ORF: open reading frame; TCDB: Transporter classification database; E: E-value

From genome analysis of *B. mongoliense* DSM 21395, 57 genes encoding for glycoside hydrolases (GH) were identified (Fig. 1a), among which 10 encode for GH family GH3, 12 for GH13 and 2 for GH33. Seventy and 19 genes encoding for glycoside transferases (GT) and carbohydrate-binding modules (CBM) were found, respectively (Fig. 1b and c). Six genes encoding for enzymes involved in HMO or BMO degradation were highlighted: one cytoplasmic β-hexosaminidase (GH20), one cytoplasmic α-L-fucosidase (GH95), two cytoplasmic β-galactosidases (GH2 and GH42) and two extracellular α-sialidases (GH33) (Fig. 2). In addition, 36 genes were predicted to be involved in sugars transport, among which 12 encode for HMO or BMO monosaccharides and oligosaccharides (Fig. 3b). Putative transporters belonging to the TCDB family 3.A.1.24, 3.A.1.25 and 3.A.1.2.20 provided glucose transport. Putative transporters 3.A.1.1.18, 3.A.1.2.22 and 3.A.1.1.48 assured transport of GlcNAc, oligosaccharides and lacto-N-biose or galacto-N-biose, respectively.

From genome analysis of *B. crudilactis* LMG 23609, 48 genes encoding for GH were identified (Fig. 1a), among which 3 encode for GH family GH3 and 12 for GH13. Sixty-eight and 19 genes encoding for GT and CBM were found, respectively (Fig. 1b and c). Two genes encoding for cytoplasmic β-galactosidases (GH2 and GH42) were highlighted (Fig. 2). From the *B. mongoliense* DSM 21395 genome, 28 genes were predicted to be involved in sugars transport, among which eight encode for HMO or BMO monosaccharides and oligosaccharides (Fig. 3b). Putative transporters belonging to the TCDB family 2.A.1.68.1 and 2.A.7.5.5 provided glucose transport. Putative transporters 2.A.2.2.3 and 3.A.1.1.48 assured transport of galactose and lacto-N-biose or galacto-N-biose, respectively.

### Growth of *B. mongoliense* strain FR/49/f/2

The highest counts were observed on MRS2-G and MRS2-Wh media (8.6 ± 0.8 log cfu/mL and 8.3 ± 1.0 log cfu/mL, respectively) and the lowest count on MRS2–3′SL medium (6.7 ± 0.3 log cfu/mL). No growth was observed on MRS2 medium (6.0 ± 0.1 log cfu/mL) (Table 1).

**Table 1:** Counts of *B. mongoliense* after incubation in MRS2, MRS2-G, MRS2-Wh and MRS2–3′SL media

	Final concentrations after 48 h incubation (log cfu/ml)
MRS2	6.0 ± 0.1
MRS2-G	8.6 ± 0.8
MRS2-Wh	8.3 ± 0.8
MRS2–3′SL	6.7 ± 0.3

### Effect of CFSM on *E. coli* O157:H7 virulence gene expression

**Fig. 4:**
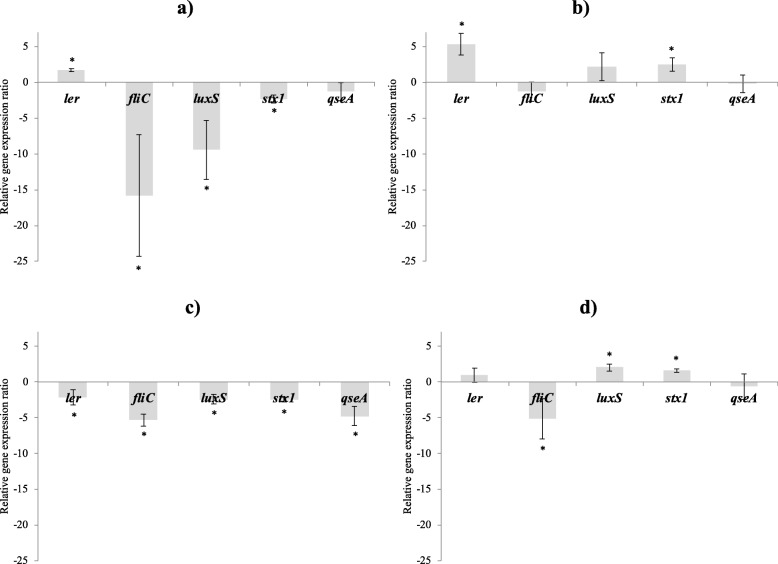
Effects of tested CFSM on *E. coli* O157:H7 virulence expression. Virulence modulations observed with CFSM obtained from MRS2 (**a**), MRS2-G (**b**), MRS2-Wh (**c**) and MRS2–3′SL (d) media fermented by *B. mongoliense* after 4 h of incubation. Gene expression ratios of *E. coli* O157:H7 were normalised to the housekeeping gene *gnd* and compared with those of the unfermented media. Values are expressed as the average of at least 3 independent replicates, bars representing standard errors. Negative values (lower than − 2) represent down-regulation of genes and positive values (higher than 2) represent up-regulation of genes. The ratio presented in this figure were considered with a calibrator ratio resulting from a control population (without modulation of gene expression). To determine a significant modulation of the ratio before and after contact with CFSM, a Wilcoxon test for paired samples was performed where *p* < 0.05 was considered as significant. ^*^P < 0.05

After an incubation of 4 h, the CFSM had no negative impact on growth. The average OD of *E. coli* O157:H7 at 600 nm after 4 h exposure was around 0.91 ± 0.14, while it was initially at 0.05 ± 0.03. The CFSM obtained from MRS2-Wh medium induced statistically significant down-regulation of *ler, fliC*, *luxS*, *stx1* and *qseA* genes (− 2.2, − 5.3, − 2.4, − 2.5 and − 4.8, respectively; *P* < 0.05) (Fig. 4c). The CFSM from MRS2 medium induced a significant down-regulation of *fliC*, *luxS* and *stx1* genes (− 15.8, − 9.5 and − 2.3, respectively; *P* < 0.05) (Fig. 4a). A significant increase of *ler* gene expression was observed with this same medium, but too low to be biologically meaningful (1.7; *P* < 0.05). CFSM from MRS2-G medium tended to over-express the *luxS* gene (2.2) and two significant up-regulations of *ler* and *stx1* genes (5.3 and 2.5, respectively; *P* < 0.05) were observed (Fig. 4b). A significant down-regulation of the *fliC* gene was noted with the CFSM from MRS2–3′SL medium (− 5.2; *P* < 0.05) (Fig. 4d). A significant up-regulation was also observed with the *luxS* gene (2.0; *P* < 0.05), while the significant increase of the *stx1* gene expression was considered too low to be biologically meaningful (1.6; *P* < 0.05). The details of the cycle threshold values corresponding to the effects of CFSM on *E. coli* O157:H7 genes expression are available in the Additional file 1: Table S1.

### Effect of CFSM on *S.* Typhimurium virulence gene expression

**Fig. 5:**
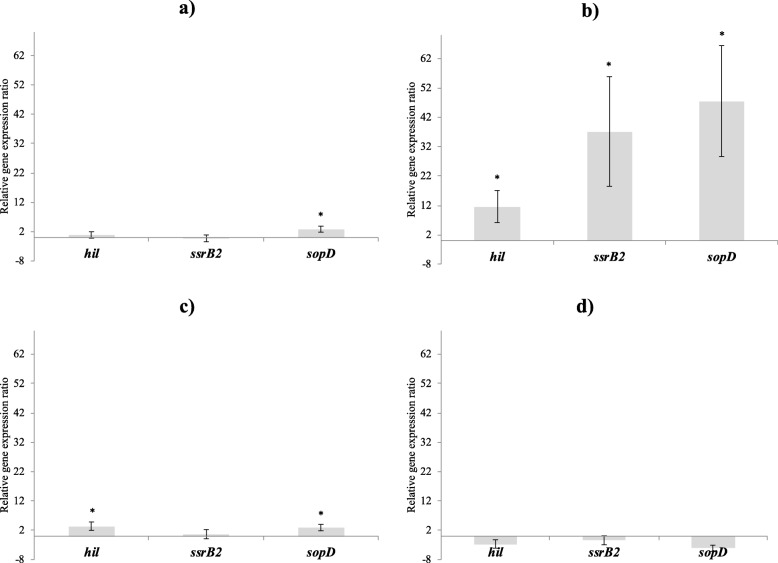
Effects of tested CFSM on *S.* Typhimurium virulence expression. Virulence modulations observed with CFSM obtained from MRS2 (**a**), MRS2-G (**b**), MRS2-Wh (**c**) and MRS2–3′SL (d) media fermented by *B. mongoliense* after 4 h of incubation. Gene expression ratios of *S.* Typhimurium were normalised to the housekeeping gene *gmk* and compared with those of the unfermented media. Values are expressed as the average of at least 3 independent replicates, bars representing standard errors. Negative values (lower than − 2) represent down-regulation of genes and positive values (higher than 2) represent up-regulation of genes. The ratio presented in this figure were considered with a calibrator ratio resulting from a control population (without modulation of gene expression). To determine a significant modulation of the ratio before and after contact with CFSM, a Wilcoxon test for paired samples was performed where *p* < 0.05 was considered as significant. ^*^P < 0.05

After the 4 h incubation, the OD measurements showed no negative impact on growth. The average OD of *S.* Typhimurium at 600 nm and after 4 h of exposure was 0.90 ± 0.04, while it was initially at 0.05 ± 0.02. CFSM obtained from MRS2 medium induced a significant up-regulation of the *sopD* gene (2.8; *P* < 0.05) (Fig. 5a) while the MRS2-Wh CFSM led to significant up-regulation of *sopD* and *hil* genes (2.9 and 3.4, respectively; *P* < 0.05) (Fig. 5c).

Three significant up-regulations of *hil*, *ssrB2* and *sopD* genes (11.6, 37.2, 47.5, respectively; *P* < 0.05) were noted for the CFSM from MRS2-G medium (Fig. 5b). A trend for down-regulation of *hil* and *sopD* genes (− 2.7 and − 4.2, respectively) was observed with CFSM obtained from MRS-3′SL medium (Fig. 5d). The details of the cycle threshold values corresponding to the effects of CFSM on *S.* Typhimurium genes expression are available in the Additional file 2: Table S2.

## Discussion

As explained previously, *B. mongoliense* FR/49/f/2 and *B. crudilatis* FR/62/b/3 are both from bovine origin [35] but *B. mongoliense* seems to have an enzymatic arsenal more sophisticated to digest BMO compared to *B. crudilactis*. In the *B. mongoliense* DSM21395 genome, many genes encoded enzymes belonging to the GH families involved in complex carbohydrates degradation, such as GH2 (β-galactosidase), GH3 (glucosidases), GH13 (amylases), GH20 (β-hexosaminidases), GH33 (α-sialidases), GH42 (β-galactosidases) and GH95 (α-L-fucosidases). Those genes were found in the *B. crudilactis* LMG 23609 genome too, with exception of those encoding for GH20 and GH95 (involved in HMO or BMO digestion) and GH33, which could be considered an advanced genetic adaptation to the intestinal environment due to its involvement in degradation of both HMO/BMO and intestinal glycoconjugates such as mucin [37]. In addition, with its 14 potential genes involved in milk sugars transport, half of them being related to transfer of oligosaccharides, the transport system of *B. mongoliense* DSM21395 seems more efficient compared to *B. crudilactis* LMG 23609. Regarding *B. crudilactis* LMG 23609, eight potential genes are involved in milk sugar transport and most of them are involved in the transport of monosaccharides such as glucose and galactose. From the analysis of the TCDB families, *B. mongoliense* DSM21395 and *B. crudilactis* LMG 23609 could be able to transport LNB. However, LNB is a disaccharide associated with HMO rather than BMO [22]. Indeed, for most of the HMO, Gal and GlcNAc are linked to β1–3 liaison (corresponding to LNB in HMO), contrarily to BMO, for which Gal and GlcNAc are linked to β1–4 liaison (related to LacNac in BMO). In addition, the very high E-values observed with transporters belonging to the TCDB family 3.A.1.1.48 indicate a very low confidence index and could suggest that another disaccharide close to LNB, such as LacNac, could be internalised by *B. mongoliense* DSM21395 or *B. crudilactis* LMG 23609.

According to the schematic representation of the BMO metabolism available in the Additional file 3: Figure S3, *B. mongoliense* could be able to digest most of the oligosaccharides found in BMO, except the sialic acid, which is supposed to stay in the external environment. In the same way, *B. mongoliense* could be able to metabolise 3′SL (NeuAcα2–3Galβ1–4Glc) using an extracellular α-sialidase. Then, the lactose (Galβ1–4Glc) could be internalised using an ABC transporter and metabolised using a cytoplasmic β-galactosidase. According to this representation, the BMO or 3′SL degradation by *B. crudilactis* seems impossible without a previous cross-feeding step involving other bifidobacteria.

*B. mongoliense* FR/49/f/2 showed good growth on media supplemented with either whey or glucose, whey contribution making no significant difference compared to glucose. In addition, whey is rich in lactose [38], a carbohydrate source easily consumed by bifidobacteria [39], and in several BMO (encompassing 3′SL) [25]. Finally, the important growth observed with the medium supplemented with whey is probably due to the high concentration in lactose, glucose and potentially BMO. Interestingly, the strain *B. mongoliense* FR/49/f/2 grew on medium containing 3′SL as the main carbohydrate source, in comparison to the absence of growth observed on medium without any carbohydrate source. According to *B. mongoliense* DSM21395 genome analysis, this growth could result from the expression of the enzymes cleaving BMO or 3′SL bonds, such as α-sialidases, β-galactosidases and β-hexosaminidases. Genes involved in BMO degradation are also present in other bifidobacteria such as *B. bifidum* subsp. *infantis* and *B. bifidum* [36, 39, 40].

CFSM issued from lactic acid bacteria or bifidobacteria contained metabolites decreasing virulence genes expression of *C. jejuni* [18], *E. coli* O157:H7 [16] and *S.* Typhimurium [17]. In our study, CFSM obtained from medium supplemented with whey and fermented by *B. mongoliense* FR/49/f/2 was able to decrease *E. coli* O157:H7 ATCC 43890 virulence. All the 5 tested virulence genes were significantly under-expressed (*ler*, *fliC*, *luxS*, *stx1* and *qseA*). Furthermore, with CFSM obtained from medium fermented without any glucose, three genes were significantly down-regulated (*fliC*, *luxS* and *stx1*). In the study of Bondue et al. [41], CFSM obtained from medium supplemented with 3′SL and fermented by *B. bifidum* BBA1 and *B. crudilactis* FR/62/B/3 presented a significant effect against *E. coli* O157:H7 ATCC 43890 virulence genes expression. Indeed, most of the virulence genes were down-regulated (*ler*, *luxS*, *stxB2* and *qseA*), except the *fliC* gene, which tended to be up-regulated. These results are different from what is observed in this study with *B. mongoliense* (significant down-regulation of *fliC* gene and significant up-regulation of *luxS* and *stx1* genes). According to the genome of *B. mongoliense*, including many genes encoding enzymes from the GH family, metabolites produced in medium supplemented with 3′SL and fermented by *B. mongoliense* FR/49/f/2 could be different than those produced by *B. crudilactis* FR/62/B/3 and *B. bifidum* BBA1. Also, the down-regulation of the *fliC* gene while other virulence genes were up-regulated has already been demonstrated in other studies [42–44]. The media MRS2-G and MRS2-Wh are rich in carbohydrates, and fermentation products such as lactate and acetate are synthesised. To prevent an inhibition of pathogenic bacteria growth due to media acidification, all CFSM were neutralised before testing them with *E. coli* or *S.* Typhimurium as described previously [16, 41].

After contact with the CFSM, *S.* Typhimurium ATCC 14028 virulence gene expression was modulated, but not always in the same way as *E. coli* O157:H7 ATCC 43890. The CFSM from medium supplemented with glucose gave the same kind of regulation for both pathogens, and all the tested genes were over-expressed with *S.* Typhimurium (*hil*, *ssrB2* and *sopD*). CFSM from media without any glucose or supplementation with whey down-regulated *E. coli* virulence genes, while these CFSMs induced mainly up-regulations of *Salmonella* virulence genes (*hil* and *sopD*). The only trend for down-regulation of *Salmonella* virulence genes was observed with CFSM supplemented with 3′SL (*hil* and *sopD* genes). The metabolites obtained from the fermentation of 3′SL by *B. mongoliense* could decrease pathogenicity of *S.* Typhimurium. This effect had already been observed with CFSM fermented by *B. bifidum* BBA1, where all the tested genes were under-expressed [41].

Little is known about the exact nature of these metabolites. These bioactive molecules could originate from the degradation of proteins such as nisin or subpeptins similar to JM4-A and JM4-B, produced by lactic bacteria and having an antimicrobial effect [45, 46]. The 3′SL or whey digestion by *B. crudilactis* FR/62/b/3 or *B. mongoliense* FR/49/f/2 has an antivirulent effect on *E. coli* O157:H7 and *S.* Typhimurium [41] and these bioactive molecules could be related to some carbohydrate residues, if they are obtained from carbohydrate metabolism. β-galactosidase genes, necessary for 3′SL degradation, have been identified in the *B. crudilactis* LMG 23609 genome but do not seem sufficient to easily metabolise the BMO. The enzymatic machinery proposed for BMO degradation identified in the *B. mongoliense* DSM21395 genome is more sophisticated with the putative presence of β-galactosidases, but also with α-sialidases and β-hexosaminidases. This highlights the putative capacity of *B. mongoliense* to metabolise the 3′SL and other complex BMO. The carbohydrate residues issuing from this degradation, such as sialic acid, glc, gal or GlcNac, could be involved in this antivirulent effect (Figure S3). The potential metabolites from 3′SL or whey degradation are probably different from those mentioned above and could explain why the 3′SL and whey fermentation by *B. mongoliense* FR/49/f/2 CFSM did not have the same antivirulence effect on *E. coli* O157:H7 compared to *B. crudilactis* FR/62/b/3 CFSM. Information is lacking about the exact nature of these molecules (peptides or glucidic residues), and a size-exclusion chromatography in association with a mass spectrometry could contribute to further identifying them.

## Conclusions

In conclusion, according to the genome analysis of *B. mongoliense* DSM21395 associated to the growth of *B. mongoliense* FR/49/f/2 on media supplemented with whey or 3′SL, *B. mongoliense* presents a genotype more adapted to the digestion of BMO compared to *B. crudilactis*. These two different kinds of metabolism observed for these two bifidobacteria species from bovine origin could have an impact on the nature of produced residual metabolites, which could differ in their influence on the virulence expression of pathogens. CFSM obtained from medium supplemented with whey and fermented by *B. mongoliense* FR/49/f/2 presented the most interesting effect by decreasing the virulence expression of the five tested genes of *E. coli* O157:H7 (*ler*, *fliC*, *luxS*, *stx1* and *qseA*). For *S.* Typhimurium, the CFSM obtained after 3′SL fermentation presented the most interesting effect by decreasing 2 of the 3 tested genes (*hil* and *sopD*). According to this study, *B. mongoliense* FR/49/f/2 could be a potential probiotic, which associated to BMO, could maintain the young child’s gastrointestinal health through a synbiotic effect.

## Methods

### Genome analysis

The genomes of the two reference strains, *B. crudilactis* LMG 23609 and *B. mongoliense* DSM 21395 are available on GenBank using accession number JHAL00000000 and JGZE00000000, respectively [35, 36, 47]. The encoded genomes were submitted to homology search against the CAZy database (PMID: 24270786) using the MEGAnnotator software (REF PMID: 26936607). Transporters’ specificity was predicted by means of the Transporter Classification DataBase (TCDB) (REF PMID: 26546518). Cellular localisation of putative HMO/BMO degradation genes was defined based on the PSORTb v3.0 web server (REF PMID: 20472543).

### Bacterial strains and growth conditions

*B. mongoliense* FR/49/f/2 and *B. crudilactis* FR/62/b/3 have been isolated from Saint-Marcellin, a raw cow milk cheese from the Vercors area (France). These strains as well as EHEC strain O157:H7 ATCC 43890 and *S. enterica* serovar Typhimurium strain ATCC 14028 were stored and grown following the experimental protocol described previously by Bondue et al. [41].

Four media with different carbohydrate sources were used: a medium without any glucose (MRS2), a reference medium with glucose (MRS2-G) [48], a medium with a mix of glucose and whey as a source of BMO (MRS2-Wh), and a medium with 3′SL (MRS2–3′SL) as the main source of carbohydrate (Table 2). The yeast extract, peptone of casein and glucose were provided by the Oxoid firm (Temse, Belgium). The tween 80 was provided by Sigma-Aldrich Laboratory (Diegem, Belgium) and the K_2_HPO_4_, KH_2_PO4, NaCl, MnSO_4_^.^H_2_O, MgSO_4_^.^7H_2_O, FeSO_4_^.^7H_2_O and cysteine by Merck Laboratory (Overijse, Belgium). Sweet whey (12°D) was collected at the beginning of a curdling process in a Belgian cheese factory (Liège area, Belgium) and frozen at − 20 °C before further use. Whey was then sterilised using double filtration (Minisart® 0.45 μm and 0.2 μm, Sartorius, Vilvoorde, Belgium). The quantities of lactose and protein in MRS2-Wh medium were estimated to be around 25 g/L and 4 g/L, respectively [38]. The 3′SL, added to MRS2–3′SL, was provided by Carbosynth Laboratory (Berkshire, UK). The purity of the 3′SL was of minimum 98%. The concentration of 0.85 g/L was chosen to be close to natural concentrations found in colostrum [23]. The experiments to obtain the concentrated CFSM containing bioactive molecules issued from *B. crudilactis* FR/62/b/3 metabolism, were elaborated by Bondue et al. [41]. *B. mongoliense* FR/49/f/2 was grown in three independent experiments on De Man, Rogosa, and Sharpe (MRS) medium (Oxoid, Hampshire, UK) supplemented with cysteine-HCl (0.5 g/L) and mupirocin (0.08 g/L) under anaerobic conditions at 37 °C for 48 h. A maximum of two successive cultures have been carried out in MRS broth to reach 8 log/mL, prior to use. Next, the cultures were used to inoculate the four previously described media to reach 6 log/mL of bifidobacteria (1% v/v) (concentration was confirmed by plating several dilutions of bifidobacteria at day 0 post inoculation). Bacterial growth was determined using viable plate counts after 48 h incubation. CFSM were obtained after two centrifugation steps at 5000 rpm (Eppendorf Centrifuge 5804, Hamburg, Germany) for 10 min. Supernatants were then sterilised by double filtration (Minisart® 0.45 μm and 0.2 μm, Sartorius, Vilvoorde, Belgium). Next, CFSM were freeze-dried (Virtis Benchtop 3.3EL, SPS Scientific, Suffolk, United Kingdom) and rehydrated with sterile distilled water to obtain a 10-fold concentration. The same treatment was applied to non-fermented culture media (controls). To prevent an inhibition of pathogenic bacteria growth due to media acidification, the pH of rehydrated CFSM was adjusted to 7 using 1 M NaOH.

**Table 2:** Composition of the modified MRS2 media adapted from Tanimomo et al. [48]

	MRS2	MRS2-G	MRS2-Wh	MRS2–3′SL
Yeast extract (g/L)	15.5	15.5	15.5	15.5
Peptone of casein (g/L)	15.5	15.5	15.5	15.5
K_2_HPO_4_ (g/L)	0.9	0.9	0.9	0.9
KH_2_PO_4_ (g/L)	0.9	0.9	0.9	0.9
NaCl (g/L)	0.009	0.009	0.009	0.009
MnSO_4_^.^H_2_O (g/L)	0.17	0.17	0.17	0.17
MgSO_4_^.^7H_2_O (g/L)	0.007	0.007	0.007	0.007
FeSO_4_^.^7H_2_O (g/L)	0.009	0.009	0.009	0.009
Tween 80 (mL/L)	0.9	0.9	0.9	0.9
Cysteine (g/L)	0.4	0.4	0.4	0.4
Glucose (g/L)	–	20	10	–
Whey (mL/L)	–	–	500	–
3′-sialyllactose (g/L)	–	–	–	0.85

### Exposure of pathogenic strains to CFSM, gene expression analysis by RT-qPCR and statistical analysis

The applied method was fully described in the previous study of Bondue et al. [41]. *E. coli* O157:H7 ATCC 43890 and *S.* Typhimurium ATCC 14028 were incubated overnight at 37 °C under agitation in LB and BHI broth, respectively. Volumes of 50 μL of homogenised cultures and 450 μL of each concentrated CFSM (fermented and unfermented) were then added to 4.5 mL of LB broth for *E. coli* and BHI broth for *S.* Typhimurium. Triplicate cultures were incubated at 37 °C for 4 h [49] on a shaker at 150 rpm. *E. coli* O157:H7 and *S.* Typhimurium were grown in LB and BHI broth alone, respectively, as controls. Bacterial growth was determined by OD measurement at 600 nm.

Cells were then collected by centrifugation at 5000 rpm for 10 min at room temperature (Eppendorf Centrifuge 5804, Hamburg, Germany) and pellets were suspended in Tris-EDTA buffer containing 1% lysozyme (Roche, Mannheim, Germany). RNA was extracted using the RNeasy® Mini Kit (Qiagen, Antwerp, Belgium) and DNA contamination was eliminated using the DNase I Recombinant RNase-free Kit (Roche Diagnostics GmbH, Mannheim, Germany). Samples were heated at 75 °C for 10 min in order to inactivate the DNase. The concentration of RNA was normalised to 100 ng/μL for *E. coli* and to 50 ng/μL for *S.* Typhimurium. The RNA was then subjected to reverse transcription polymerase chain reaction (RT-PCR) using a high-capacity cDNA Reverse Transcription Kit (Applied Biosystems, Ghent, Belgium). Synthesis of cDNA was performed in a Mastercycler Gradient Thermocycler (Flexigene, Cambridge, United Kingdom) under the following conditions: 25 °C for 10 min, 37 °C for 120 min, 85 °C for 5 min and a cooling step at 4 °C. A no-RT control was made to confirm the absence of DNA contamination in each sample.

To highlight the effects of filtrated supernatants on virulence gene expressions of *E. coli* O157:H7 ATCC 43890, the expression of *ler*, *fliC*, *stx1*, *luxS*, and *qseA* genes was determined using qPCR with *gnd* as a reference housekeeping gene [50, 51]. For *S.* Typhimurium ATCC 14028, the virulence expressions of *hilA*, *ssrB2* and *sopD* genes were assessed using *gmk* as a reference housekeeping gene [52]. Quantitative PCR amplification was performed using the GoTaq® qPCR Master Mix (Promega, Leiden, Netherlands) and using the Light Cycler 480 (Roche Diagnostics, Mannheim, Germany). The primers were synthesised by Eurogentec (Liège, Belgium) and had been designed and validated in previous studies [37]. The qPCR conditions for *E. coli* were: initial denaturation at 95 °C for 3 min; denaturation, annealing and elongation repeated 45 times: 95 °C for 15 s, 58 °C for 30 s and 72 °C for 45 s; melting curve program of 60–95 °C with a heating rate of 0.1 °C/s. The qPCR conditions for *S.* Typhimurium were: denaturation at 95 °C for 10 min; 40 cycles of amplification and quantification at 95 °C for 30 s, 56 °C for 30 s and 72 °C for 30 s; melting curve program of 60–95 °C with a heating rate of 0.1 °C/s. The experiments were replicated three independent times and special attention was given to the controls to exclude any potential effect observed from the non-fermented media as described previously [41, 49]. The relative changes in gene expression were calculated using the Pfaffl formula [53]: ratio = (virulence gene Efficiency)^Ct unfermented – Ct fermented^ / (reference gene Efficiency)^Ct unfermented – Ct fermented^, with Efficiency = 10^(− 1/slope)^. The ratio obtained was considered with a calibrator ratio resulting from a control population (without modulation of gene expression). To determine a significant modulation of the ratio before and after contact with CFSM, a Wilcoxon test for paired samples was performed where *p* < 0.05 was considered as significant.

## Supplementary information

**Additional file 1: Table S1.** Cycle threshold values corresponding to the effects of tested CFSM on *E. coli* O157:H7 genes expression.

**Additional file 2: Table S2.** Cycle threshold values corresponding to the effects of tested CFSM on *S.* Typhimurium genes expression.

**Additional file 3: Figure S3.** Schematic representation of BMO and 3′SL metabolism by *B. mongoliense* and *B. crudilactis*.

## Data Availability

All data are available from the corresponding author on reasonable request.

## Abbreviations

3′SL: 3′-sialyllactose
AE: Attaching and effacing
BHI: Brain heart infusion
BMO: Bovine milk oligosaccharide
CBM: Carbohydrate-binding module
CFSM: Cell-free spent medium
E: E-value
EHEC: Enterohaemorrhagic *Escherichia coli*
FOS: Fructo-oligosaccharide
fuc: Fucose
gal: Galactose
GH: Glycoside hydrolase
GT: Glycoside transferase
glc: Glucose
GlcNAc: N-acetylglucosamine
GOS: Galacto-oligosaccharide
HIV: Human immunodeficiency virus
HMO: Human milk oligosaccharide
LacNac: N-acetyllactosamine
LB: Luria Bertani
LNB: Lacto-N-biose
LNnt: Lacto-N-neotetraose
LNT: Lacto-N-tetraose
MRS: De Man, Rogosa and Sharpe
NeuAc: N-acetylneuraminic acid or sialic acid
NeuGc: N-glycolylneuraminic acid
OD: Optical density
ORF: Open reading frame
TCDB: Transporter classification database
T3SS: Type III system secretion
