# Supplementary material for: Bifidobacterium mongoliense genome seems particularly adapted to milk oligosaccharide digestion leading to production of antivirulent metabolites
Source: BMC Microbiol. 2020 May 7;20:111. doi: 10.1186/s12866-020-01804-9 (PMC7206731; doi:10.1186/s12866-020-01804-9)
Supplement: Supplementary file 1 — Additional file 1: Table S1. Cycle threshold values corresponding to the effects of tested CFSM on E. coli O157:H7 genes expression. [file 12866_2020_1804_MOESM1_ESM.pdf]

Table S1 Cycle threshold values corresponding to the effects of tested CFSM on *E. coli* O157:H7 genes expression

Gene	PCR efficiency[a] (%)	LB control[b]	Test supernatants from unfermented media[c]				CFSM from fermented media by *B. mongoliense*[d]			
			MRS2 ~~G~~	MRS2	MRS2-Wh	MRS2-3'SL	MRS2 ~~G~~	MRS2	MRS2-Wh	MRS2-3'SL
gnd	98.5	16.5 ± 0.9	21.3	21.2	25.8	24.3	21.8 ± 0.4	20.9 ± 0.7	25.3 ± 0.7	22.2 ± 1.1
ler	99	17.9 ± 0.8	24.9	27.2	28.9	27.8	24.6 ± 0.3	24.6 ± 0.7	29.2 ± 0.9	25.1 ± 1.3
fliC	98	20.4 ± 2.3	17.8	21.9	29.2	22.6	21.7 ± 1.2	22.3 ± 1.0	31.2 ± 0.9	22.3 ± 1.7
luxS	100	23.7 ± 2.2	26.7	28.8	33.8	32.2	30.0 ± 0.9	27.4 ± 1.2	34.5 ± 0.9	29.3 ± 1.2
stx1	98	28.9 ± 1.7	26.1	28.2	31.5	30.1	27.7 ± 0.5	26.9 ± 0.9	32.3 ± 0.9	27.3 ± 0.9
qseA	99	20.1 ± 1.3	24.5	25.0	28.9	27.5	25.8 ± 0.3	24.7 ± 1.0	30.6 ± 0.9	25.7 ± 0.3

[a]PCR efficiency: $E = [(10^{(-1/slope)})/2] \times 100\ \%$

[b]*E. coli* O157:H7 grown in LB broth for 4 h.

[c]*E. coli* O157:H7 grown in LB broth supplemented with unfermented culture media CFSM for 4 h.

[d]*E. coli* O157:H7 grown in LB broth supplemented with fermented concentrated culture media CFSM from *B. mongoliense* for 4 h.
