# Supplementary material for: Bifidobacterium mongoliense genome seems particularly adapted to milk oligosaccharide digestion leading to production of antivirulent metabolites
Source: BMC Microbiol. 2020 May 7;20:111. doi: 10.1186/s12866-020-01804-9 (PMC7206731; doi:10.1186/s12866-020-01804-9)
Supplement: Supplementary file 2 — Additional file 2: Table S2. Cycle threshold values corresponding to the effects of tested CFSM on S. Typhimurium genes expression. [file 12866_2020_1804_MOESM2_ESM.pdf]

Table S2 Cycle threshold values corresponding to the effects of tested CFSM on *S.* Typhimurium genes expression

Gene	PCR efficiency[a] (%)	BHI control[b]	Test supernatants from unfermented media[c]				CFSM from fermented media by *B. mongoliense*[d]			
			MRS2 ~~G~~	MRS2	MRS2-Wh	MRS2-3'SL	MRS2 ~~G~~	MRS2	MRS2-Wh	MRS2-3'SL
gmk	100	25.3 ± 1.6	21.0	20.2	25.2	28.7	23.9 ± 1.1	26.9 ± 0.7	24.0 ± 1.1	25.9 ± 1.8
hilA	91	31.0 ± 0.3	27.2	25.9	34.2	34.2	30.6 ± 1.3	30.8 ± 1.9	28.9 ± 1.2	31.8 ± 1.5
ssrB2	115	30.9 ± 0.2	28.0	27.8	34.5	33.7	30.7 ± 1.0	29.9 ± 1.4	30.8 ± 1.2	31.7 ± 1.2
sopD	91	29.9 ± 0.1	27.8	27.6	34.2	33.0	30.1 ± 1.0	29.9 ± 1.7	29.1 ± 0.4	30.8 ± 1.2

[a]PCR efficiency: $E = [(10^{(-1/slope)})/2] \times 100$ %

[b]*S.* Typhimurium grown in BHI broth for 4 h.

[c]*S.* Typhimurium grown in BHI broth supplemented with concentrated supernatants from culture media unfermented for 4 h.

[d]*S.* Typhimurium grown in BHI broth supplemented with concentrated supernatants from culture media fermented *B. mongoliense* for 4 h.
