# Supplementary figures and images for: Bifidobacterium mongoliense genome seems particularly adapted to milk oligosaccharide digestion leading to production of antivirulent metabolites
Source: BMC Microbiol. 2020 May 7;20:111. doi: 10.1186/s12866-020-01804-9 (PMC7206731; doi:10.1186/s12866-020-01804-9)

Figure S3. Schematic representation of BMO and 3'SL metabolism by *B. mongoliense* and *B. crudilactis*.

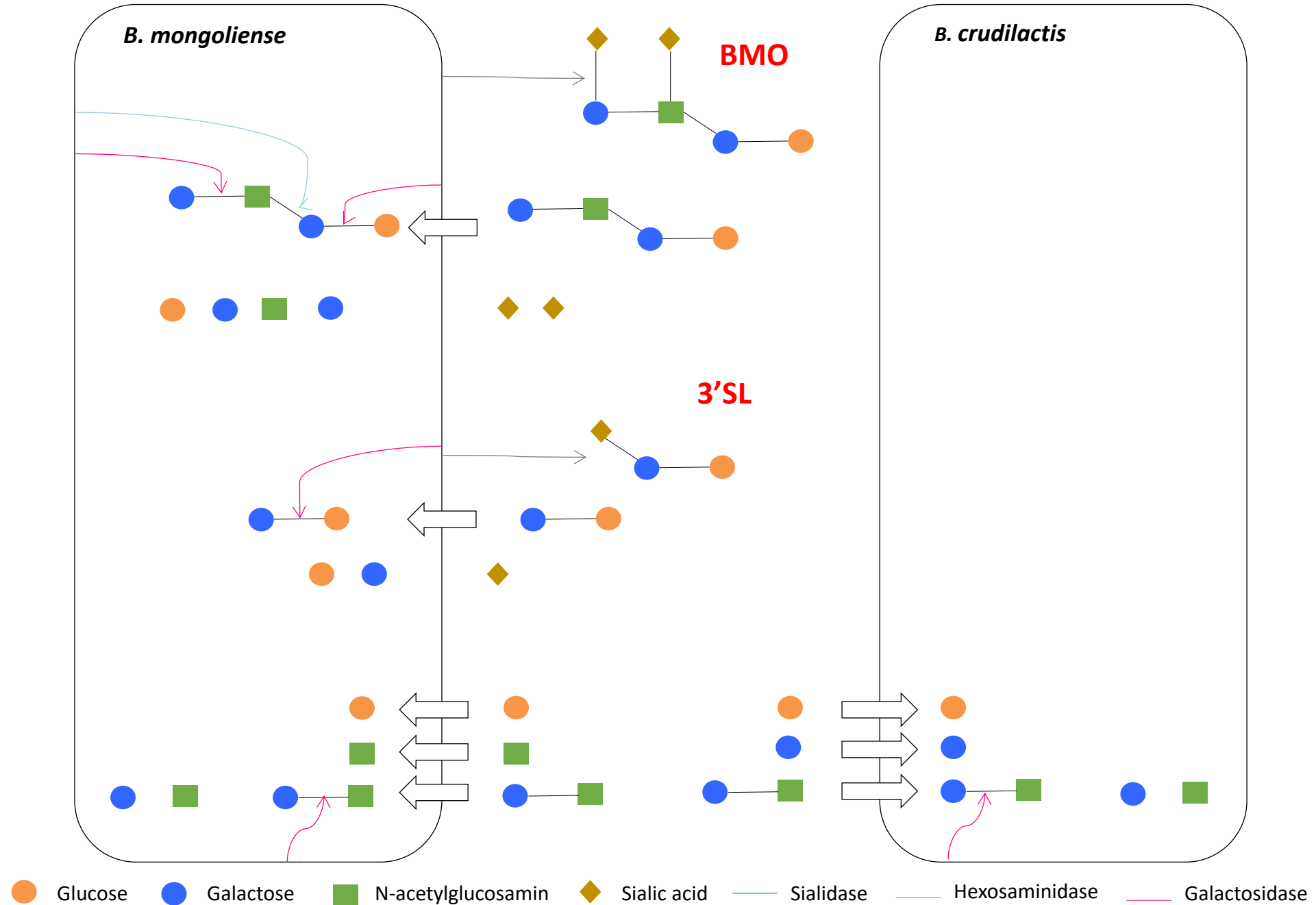

Supplement: Supplementary file 3 — Additional file 3: Figure S3. Schematic representation of BMO and 3′SL metabolism by B. mongoliense and B. crudilactis. [file 12866_2020_1804_MOESM3_ESM.pdf]
